# Multimodal quantitative magnetic resonance imaging analysis with individualized postprocessing in patients with drug-resistant focal epilepsy and conventional visual inspection negative for epileptogenic lesions

**DOI:** 10.6061/clinics/2019/e908

**Published:** 2019-07-17

**Authors:** Lucas Giansante Abud, Tonicarlo Rodrigues Velasco, Carlos Ernesto Garrido Salmon, Americo Ceiki Sakamoto, Thiago Giansante Abud, Rodrigo Antonio Pessini, Daniel Giansante Abud, João Pereira Leite, Antonio Carlos dos Santos

**Affiliations:** I Divisao de Neurorradiologia, Faculdade de Medicina de Ribeirao Preto, Universidade de Sao Paulo, Ribeirao Preto, SP, BR; II Departamento de Neurologia, Faculdade de Medicina de Ribeirao Preto, Universidade de Sao Paulo, Ribeirao Preto, SP, BR; III Departamento de Fisica e Matematica, Faculdade de Filosofia, Ciencias e Letras de Ribeirao Preto, Universidade de Sao Paulo, Ribeirao Preto, SP, BR; IV Departamento de Diagnostico por Imagem, Escola Paulista de Medicina, Universidade Federal de Sao Paulo, Sao Paulo, SP, BR; V Divisao de Ciencias da Imagem e Fisica Medica, Faculdade de Medicina de Ribeirao Preto, Universidade de Sao Paulo, Ribeirao Preto, SP, BR

**Keywords:** Drug-Resistant Epilepsy, Magnetic Resonance Imaging, Multimodal Imaging, Computer-Assisted Image Processing

## Abstract

**OBJECTIVES:**

Approximately one-third of candidates for epilepsy surgery have no visible abnormalities on conventional magnetic resonance imaging. This is extremely discouraging, as these patients have a less favorable prognosis. We aimed to evaluate the utility of quantitative magnetic resonance imaging in patients with drug-resistant neocortical focal epilepsy and negative imaging.

**METHODS:**

A prospective study including 46 patients evaluated through individualized postprocessing of five quantitative measures: cortical thickness, white and gray matter junction signal, relaxation rate, magnetization transfer ratio, and mean diffusivity. Scalp video-electroencephalography was used to suggest the epileptogenic zone. A volumetric fluid-attenuated inversion recovery sequence was performed to aid visual inspection. A critical assessment of follow-up was also conducted throughout the study.

**RESULTS:**

In the subgroup classified as having an epileptogenic zone, individualized postprocessing detected abnormalities within the region of electroclinical origin in 9.7% to 31.0% of patients. Abnormalities outside the epileptogenic zone were more frequent, up to 51.7%. In five patients initially included with negative imaging, an epileptogenic structural abnormality was identified when a new visual magnetic resonance imaging inspection was guided by information gleaned from postprocessing. In three patients, epileptogenic lesions were detected after visual evaluation with volumetric fluid-attenuated sequence guided by video electroencephalography.

**CONCLUSION:**

Although quantitative magnetic resonance imaging analyses may suggest hidden structural lesions, caution is warranted because of the apparent low specificity of these findings for the epileptogenic zone. Conversely, these methods can be used to prevent visible lesions from being ignored, even in referral centers. In parallel, we need to highlight the positive contribution of the volumetric fluid-attenuated sequence.

## INTRODUCTION

Epilepsy is defined as a brain disease defined by any of the following conditions: 1) at least two unprovoked (or reflex) seizures occurring >24h apart; 2) one unprovoked (or reflex) seizure and a probability of further seizures similar to the general recurrence risk (at least 60%) after two unprovoked seizures, occurring over the next 10 years; and 3) diagnosis of an epilepsy syndrome 1. Epilepsy affects more than 50 million people worldwide and is considered a public health problem 2.

Symptomatic focal epilepsies account for approximately 60% of all cases, and approximately one-third of these patients present with drug-resistant epilepsy despite adequate trials of two tolerated, appropriately chosen and used antiepileptic drug regimens. In selected cases, epilepsy surgery may be indicated for seizure control 3.

Magnetic resonance imaging (MRI) has emerged as an indispensable tool for the preoperative localization of epileptogenic lesions in people with drug-resistant epilepsy 3. Important issues to consider during MRI evaluation of the epileptic patient, which may increase sensitivity for detecting structural abnormalities, include the use of specific protocols, expertise of the neuroradiologist, and use of the strongest magnetic field available 4,5.

Approximately one-third of candidates for epilepsy surgery have no visible abnormalities on conventional MRI 3. In other words, even if an epileptogenic lesion exists, it may be invisible on conventional MRI sequences, especially when subtle focal cortical dysplasia (FCD) is present. This is extremely discouraging, as patients without visible lesions on MRI are also less likely to be referred for surgery and, following surgery, have a less favorable prognosis than those with visible lesions on MRI 6.

Quantitative MRI techniques involving individualized postprocessing have been applied to this group of patients and may increase sensitivity to detect focal abnormalities 7. These methods are being used to improve the detection of epileptogenic substrates, especially when conventional MRI is inconclusive. Despite much published research on MR neuroimaging, advanced MRI techniques, and quantitative MRI analyses in patients with drug-resistant neocortical focal epilepsy (DRNFE) and nonlesional epilepsy (NLE), the literature is scarce regarding studies that evaluate several quantitative methods and advanced sequences in the same cohort of patients, and the few available studies have largely been retrospective.

This study sought to evaluate, in a referral center for epilepsy surgery, the utility of several quantitative MRI methods with individualized postprocessing in a cohort of patients with DRNFE, who were potential surgical candidates and had been deemed nonlesional by 3-Tesla (3T) conventional MRI.

## METHODS

This was a prospective study of 46 patients (24 women) with suspected neocortical focal epileptic seizures who were potential candidates for surgery. All patients had NLE on conventional MRI. Our routine MRI protocol for epilepsy includes an axial turbo spin-echo T2-weighted sequence; a coronal turbo spin-echo T2-weighted sequence perpendicular to the long axis of the hippocampus; a coronal fluid-attenuated inversion recovery (FLAIR) image perpendicular to the long axis of the hippocampus; an axial T2*-weighted gradient-echo sequence or susceptibility-weighted imaging (SWI); and a T1 magnetization-prepared rapid acquisition gradient echo (MP-RAGE). High-resolution isotropic 3D-FLAIR was also performed to aid visual inspections. This sequence was not initially part of the protocol and was performed in 16 patients (16/46 or 34.8%).

All procedures were in accordance with the ethical standards of the institutional and/or national research committee and with the 1964 Helsinki Declaration and its later amendments or comparable ethical standards. Informed consent was obtained from all individual participants.

The median age was 34.6 years (range, 20-51; SD, 8.0 years), the median age at the onset of seizures was 10.6 years (range, 0-29; SD, 7.3 years), and the median epilepsy duration was 23 years (range, 8-39 years). All MRI examinations were analyzed in the routine inspections of the imaging center and multidisciplinary meetings by physicians with expertise in epilepsy imaging.

Potential epileptogenic zones (EZs) were identified through a review of surface video-electroencephalography (VEEG) findings (Nihon-Koden). Patients were then classified as having a suspected location for the focus (SLF) or no suspected location for the focus (NSLF). Briefly, patients who had symptoms and electrophysiological findings indicative of a specific region/lobe and side of the brain were classified as SLF, as were patients with relatively specific regionalization (e.g., to the anterior frontal region, mesial frontal region, or temporooccipital region) despite no lateralization. All other patients were considered NSLF. Overall, 31 patients (67.4%) were classified as SLF, and 15 (32.6%) were classified as NSLF.

All patients underwent a new 3T-MRI scan between March 2010 and June 2016 (Achieva Duo-scanner, Philips Medical Systems, Best, Netherlands). This so-called advanced protocol included four sequences.

The first sequence was a sagittal 3D T1-weighted magnetization-prepared rapid acquisition gradient echo (MP-RAGE), TR/TI/TE 2500/900/2.9 ms, flip angle 8°, matrix 240×240, field of view (FOV) 240×240, slice thickness 1.0 mm and no gap, which was used for the analysis of cortical thickness (CThk) and white and gray matter junction signal (WGJS) by specific software.

The second sequence was an axial T2 relaxometry volumetric acquisition, TR/TE 3000/(20, 40, 60, 80, 100) ms, flip angle 90°, matrix 240×180, FOV 240×180, slice thickness 3.0 mm and no gap. We calculated the T2 value for each voxel by using the linear equation: ln (S)=-TE/T2+ln (S0). Here, S and S0 represent the signal intensities for each echo time and without relaxation effect, respectively.

The third acquisition was an axial 3D proton density-weighted magnetization transfer imaging (MTI) sequence, TR/TE 7.3/3.3 ms, flip angle 8°, matrix 240×180, FOV 240×178, slice thickness 3.0 mm and no gap. In this modality, two sets of dynamic images were acquired, without and with a saturation pulse (MToff and MTon). The magnetization transfer ratio (MTR) was calculated by the equation MTR=[(MToff-MTon)/MToff]*100.

Finally, diffusion tensor imaging (DTI) axial acquisition was performed along 36 optimized noncollinear directions, TR/TE 8900/65 ms, matrix 256×256, FOV 128×128, slice thickness 2.0 mm and no gap. A single b-value of 1000 s/mm was applied, b-value measures the degree of diffusion weighting applied. Following the findings of Thivard et al. and Chen et al. 8,9, we chose mean diffusivity (MD) (the arithmetic mean of the eigenvalues of the diffusion matrix after its diagonalization) obtained directly from the scanner as the parameter of the generated maps, as it correlates best with EZ.

The control groups were extracted from the database of the imaging center of the institution where the research took place and comprised healthy volunteers with no history of neurological disorders and normal conventional MRI (Table 1). Although there was variation in N between the control groups, the variances and distributions between them were similar.

### Statistical analysis and multimodal postprocessing

For statistical analyses to be conducted among different subjects, MR images need to be spatially combined to fit a standard space created by averaging a large number of scans, known as “spatial normalization” 10,11. Other parameters used to achieve optimal results include smoothing and p-values. Smoothing consists of averaging the effect of adjacent regions for analysis, rather than being peer-to-peer. As statistical analysis is usually based on a univariate approach and comprises multiple tests, the problem of multiple comparisons must be addressed to reduce false positive results. To achieve this, in addition to smoothing, more restrictive p-values can be used to define statistical significance. To facilitate visualization and localization of abnormalities, neuroimaging software offers schematic visual identification functions using standard models and standardized brains, among other possibilities.

For analysis of CThk obtained from the 3D-T1 MP-RAGE sequence, surface-based morphometry (SBM) findings were assessed using the FreeSurfer software (Athinoula A. Martinos Center for Biomedical Imaging at Massachusetts General Hospital, Boston, MA, USA). The calculation of CThk involves a processing flow in which the surface of the pia mater and the interface between the white matter and the gray matter are identified in a technique known as SBM. The distance between these two lines is the CThk value 12. For visual identification of the significantly different regions through the QDEC tool of the FreeSurfer package, we used a recent article as a reference for our choice of smoothing, which was set at 10 mm 13.

To define the significance threshold, we used a false discovery rate (FDR)-adjusted *p*-value ≤0.05, standardized by the software, to be more restrictive than choosing a fixed standard score (z-score) and consequently achieve a more effective control of false positives.

The statistical test was the hypothesis test implemented in the QDEC tool for comparison between one subject and a group. Reported *p*-values were corrected for multiple comparisons (FDR=0.05 was selected), and permutation testing was performed, also in the QDEC tool.

Before starting the evaluation of the patient scans, 30 individuals from the overall control cohort were individually compared to the rest of the control group, and abnormalities were revealed in three individuals. Because of the lack of conformity in the literature regarding the control of false positives, we found it reasonable to expand the rate of 10% of false positives as the target to guide the choice of parameters in the statistical evaluations of the other measures. Consequently, in all evaluations, we used the same rate. Another point that supports our decision is that most studies have adopted a false positive rate higher than ours.

All other evaluations were carried out by plotting parametric maps with specific statistical inferences for a specific voxel, the voxel-based analysis (VBA) technique. We used Statistic Parametric Mapping (SPM12) software for these analyses.

WGJS maps were extracted after segmentation of the T1-weighted sequence into gray matter (GM), white matter (WM), and cerebrospinal fluid. Binarized maps were used to create a white–gray junction binary image following a previously described procedure 14. The main steps for WGJ evaluation were as follows: 1) individual WM and GM segmentation using SPM tools; 2) definition of the binary WM and GM masks, considering voxels classified with high probability (>0.9) in each tissue class; 3) definition of individual binary WGJ masks considering voxels with intensities in the interval between Mean_GM-SD_GM and Mean_WM+SD_WM; 4) convolution of individual binary WGJ masks and a unitary 3D kernel (5×5×5 voxels); and 5) Z-score maps were obtained for comparisons of each convolved patient image and the mean convolved image of the healthy control group. It bears stressing that a new mean convolved WGJ map from the group of healthy controls was created in this study.

The other maps were extracted from equations of their respective sequences. T2 and MTR maps were obtained from functions developed in-house using MINC tools. The T2 relaxometry, MTR, and MD maps were registered to an anatomical atlas space to normalize and identify those anatomical labels with significant changes. We used a B-spline interpolation as implemented in the SPM toolbox for this normalization procedure. Maps with low spatial resolution are more affected by the interpolation step in this normalization procedure.

In addition to smoothing and *p*-values, the SPM program allows limiting recognition of changes to a contiguous grouping of altered voxels (clusters); thus, small and very isolated regions (probably related to noise) are not flagged as abnormal. The *p*-values for all these maps were chosen from an uncorrected ≤0.001 or a familywise error (FWE) ≤0.05, both standardized by the software. As previously stated, *p*-values and clusters of voxels were chosen after evaluations for each measure with 20 individuals from the healthy control group conducted to achieve the same rate of false positives found in the CThk evaluation. Briefly, we then performed tests with SPM12 to find *p*-values, first using an uncorrected *p*-value ≤0.001 (less restrictive) and then an FWE-corrected *p*-value ≤0.05 (more restrictive), associated with the lowest threshold of voxel clusters that allowed reaching the same rate of false positives (two last columns of Table 1). We eliminated findings that were located outside the cerebral parenchyma and distant from the cortex, i.e., anything found in the ventricles, cisterns, sulcus, brainstem, and cerebellum, as well as in the skull bone itself and outside the skull.

Patients were evaluated for the presence of abnormalities in the individualized postprocessing of each quantitative measure. In patients classified as SLF, we considered that there was agreement between methods (electroclinical versus quantitative MRI) if the region or lobe pinpointed by MRI findings corresponded to the electroclinical source of the seizures.

### Visual evaluation of conventional MRI with specific localization guided by individualized postprocessing

The location of each abnormality in individualized postprocessing was noted to enable subsequent targeted visual inspection of conventional scans and identification of some previously undetected, subtle structural abnormalities. In cases where previously unidentified epileptogenic structural lesions were identified, we investigated what postprocessing measure allowed this detection. All sequences that are part of the routine MRI protocol for epilepsy were used at this stage.

### Critical assessment of patients' follow-up

After analyzing the quantitative data, we also performed a retrospective evaluation of all patients. In cases where previously unidentified epileptogenic structural lesions were detected during the time of data collection, we investigated whether the additional MRI sequences allowed such detection and whether the new visual inspection was guided by another method, notably VEEG.

## RESULTS

### Statistical analysis and multimodal postprocessing

**Figure 1:**
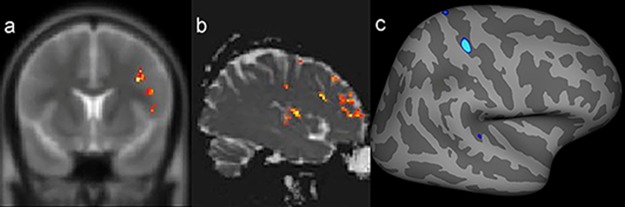
Example of a patient with a presumed epileptogenic zone in the left frontotemporal region (patient 7) that was concordant with three quantitative measures. The results of postprocessing of T2 relaxometry maps by Statistic Parametric Mapping (SPM12) software (**a:** coronal plane) and mean diffusion maps (**b:** sagittal plane), as well as cortical thickness by FreeSurfer (**c:** quadrature decoder [QDEC]), showing abnormalities notably in the left frontal lobe.

A compilation of the results of quantitative analysis through individualized postprocessing in the 46 patients is given in Table 2. A close correlation (even lobe) between the abnormal findings in at least two of these quantitative measures, which also corresponded to the electroclinical origin of the seizures, was identified in five patients in this group (5/31, 16.1%) (Figure 1).

### Visual evaluation of conventional MRI with specific localization guided by individualized postprocessing

**Figure 2:**
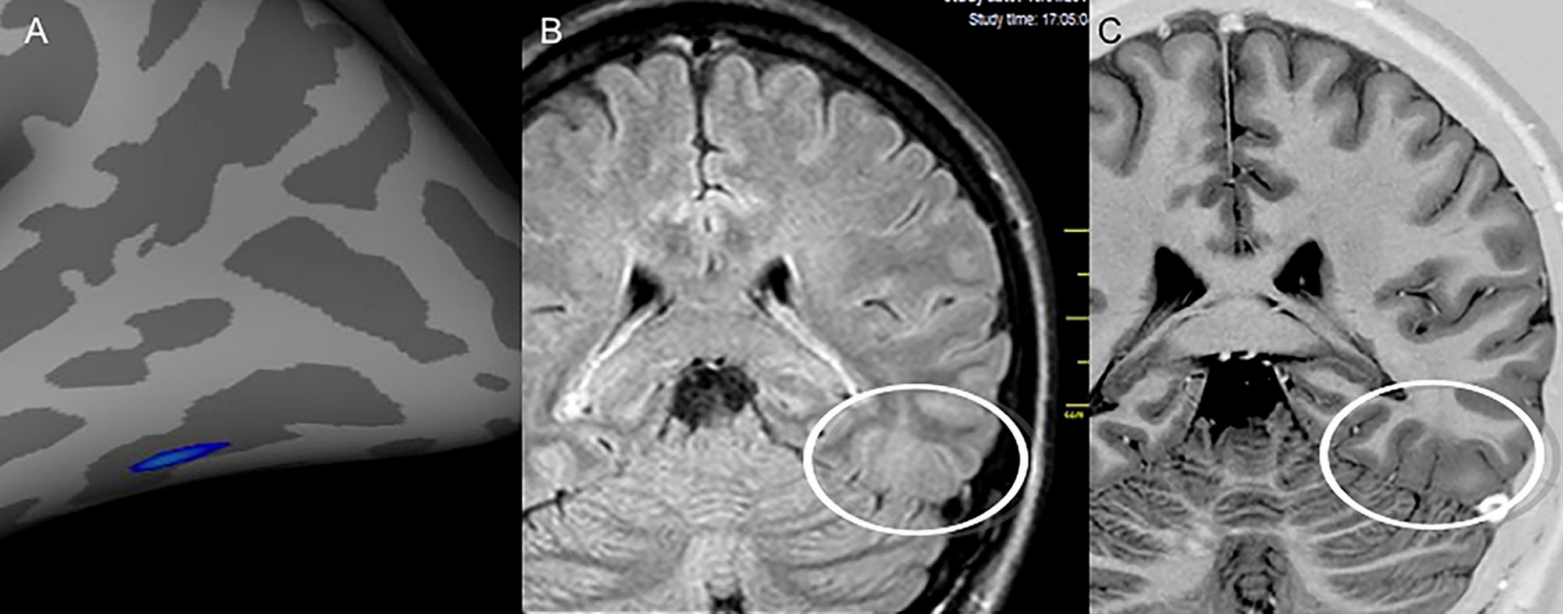
Images from patient 24 who was initially classified as magnetic resonance imaging (MRI) negative. After visual evaluation guided by postprocessing, a potentially epileptogenic structural lesion was identified in the same region considered the epileptogenic zone by electroclinical data. **A**) Results of the postprocessing of cortical thickness in the FreeSurfer software environment, showing cortical thickening in the left inferior temporooccipital transition. Nonvolumetric coronal fluid attenuation inversion recovery (FLAIR) **(B)** and T2 short tau inversion recovery (STIR) with inverted window **(C)** acquisitions showing discrete focal cortical thickening and white–gray transition blurring (white circles) in the same region identified by the quantitative analysis.

In five patients (10.9%) included in the study as NLE based on conventional MRI, an epileptogenic structural abnormality was identified on the conventional scans only after targeted visual evaluation guided by the quantitative MRI measures. All these findings were confirmed by the physicians in charge of imaging and epilepsy surgery. These patients are still in the process of being assessed for surgical referral. Three of those five patients were classified as SLF, but in one, there was disagreement between the location suggested by the electroclinical evaluation and the region of the suspected finding on MRI. The other two were considered NSLF by VEEG. SBM evaluation of the CThk was used in four patients (Figure 2), while VBA of WGJS was used in one patient.

**Figure 3:**
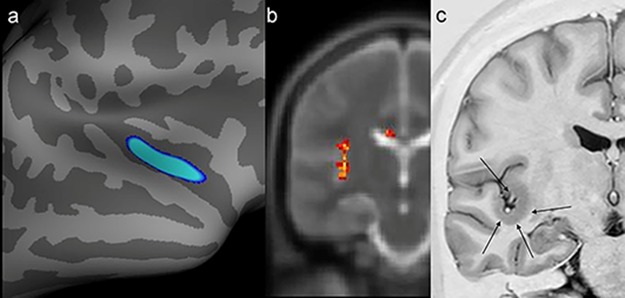
Example of a patient who switched to positive magnetic resonance imaging (MRI) status only after targeted visual evaluation guided by postprocessing (patient 19). A possible epileptogenic zone had not been regionalized in this patient with video electroencephalography. However, he exhibited abnormalities in the same region by two quantitative measures. **a)** Postprocessing of cortical thickness (quadrature decoder [QDEC]) showed temporo-insular thickening in the right hemisphere (blue image). **b)** Evaluation of T2 relaxometry maps with Statistic Parametric Mapping (SPM12) software also revealed a signal abnormality in the same location (yellow and red foci). **c)** Coronal T2 short tau inversion recovery (STIR) (inverted window) showing cortical thickening in the same region (black arrows).

In one patient, there was concordance between the findings of the SBM evaluation, VBA of T2 maps, and the structural abnormality found on visual inspection of conventional MR images (Figure 3).

### Critical assessment of patients' follow-up

**Figure 4:**
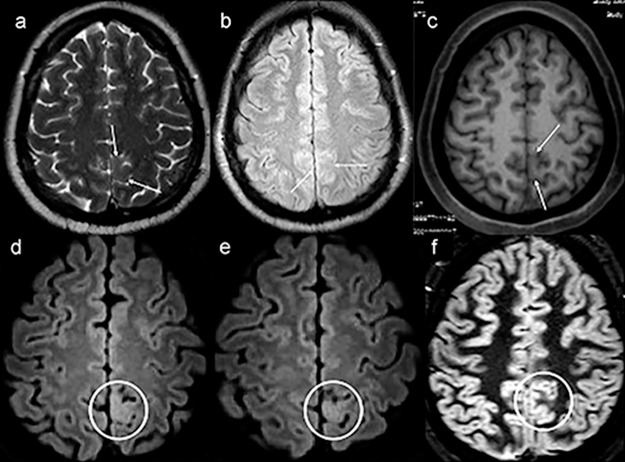
Example of a patient with no lesion (patient 39) in the first magnetic resonance imaging (MRI), exemplified by axial T2-weighted MRI **(a)**, axial nonvolumetric fluid attenuation inversion recovery (FLAIR) **(b)** and axial reconstruction of three-dimensional (3D) T1-weighted MRI **(c)**. After targeted visual evaluation guided by video-electroencephalography and 3D-FLAIR sequence, discrete focal cortical thickening and a blurring of the transition between the white and gray matter highlighted inside the white circles in the axial reconstructions were identified (**d,e**). The anatomohistopathological result was focal cortical dysplasia **(f,c,d)**. Even retrospectively, it is difficult to characterize any structural change in the routine protocol sequences for epilepsy (white arrows) **(a–c)**. The axial double inversion recovery (DIR) sequence **(f)** is also negative (white circle).

In this retrospective evaluation, three patients initially included in the study as NLE based on conventional MRI showed some structural alterations after targeted visual evaluation guided by VEEG. All underwent surgery and were diagnosed with FCD. Among these patients, 3D-FLAIR was the only sequence that allowed the identification of cortical involvement (Figure 4). In parallel, in two of the five patients in whom postprocessing guided the detection of structural alterations, the 3D-FLAIR sequence allowed for a clearer characterization of lesions.

## DISCUSSION

We used two techniques for individualized image processing, SBM in FreeSurfer software to measure CThk and VBA in SPM12 software for the other measures (WGJS, T2, MTR, and MD), with the aim of identifying cerebral abnormalities that could not be detected by visual inspection of conventional MR images in a group of patients with DRNFE, who were potential surgical candidates and had been deemed “negative” or “nonlesional” MRI. Specifically, our objective was to then evaluate the utility of these quantitative measures in this same group of consecutively enrolled patients, as the literature is very scarce in relation to the use of these MRI techniques within single cohorts, and most studies have been retrospective 7.

Postprocessing techniques require that images first be normalized in a standard space so that equivalent regions can be compared 10,11. Although there is no common statistical threshold for all studies that apply automatic and individualized postprocessing methods, a routine practice in this type of work—in which analyses are aimed at individual diagnosis, and false positive results need to be minimized—is a comparison of healthy individuals from the control group with the rest of the control group. In addition, most studies still use an arbitrary strategy to control the false positive rate; the parameters to be used (smoothing/clusters and p-values / z-scores) are initially chosen, and then the false positives are detected 7. Conversely, we decided to use a strategy that involved controlling and standardizing the false positive rate after an initial analysis, as previously described, since we considered a 10% false positive rate acceptable. As an example, some studies have worked with a detection rate of abnormalities in up to 68% of healthy individuals, demonstrating the difficulty in controlling this variable in an individual versus group evaluation 7,15.

Individual quantitative measurements detected abnormalities in any region of the brain in up to 56.4% of patients for each measurement. It is reasonable to assume that these findings are probably due to the epilepsy per se. In the subgroup of patients classified as SLF, quantitative measurements detected abnormalities in the presumed EZ at percentages ranging from 9.7% (3/31) to 31.0% (9/29) of patients with available data for each measurement. The higher increase in diagnostic yield was obtained with MD (31.0%, 9/29). However, signal abnormalities observed outside the presumed EZ were always more prevalent. Therefore, caution is recommended in the further use of these findings; if necessary, other methods, including invasive EEG recording with stereo-EEG, should be pursued for confirmation.

Previous studies of individualized postprocessing have reported good agreement between the techniques of quantitative evaluation and conventional visual analysis, as well as promising results with the use of these techniques in MRI-negative patients, indicating the potential capacity to detect epileptogenic lesions not visible on conventional imaging 7. In parallel, most studies using some of these techniques reported the same limitation found in our study regarding a more frequent rate of positive findings outside the presumed EZ; thus, this question persists as the main limitation to be approached in future studies 7,15,16. Salmenpera et al., for example, assessed four quantitative measures (T2, MTR, and two gray matter distributions) in the same group of patients with drug-resistant focal epilepsy and negative MRI through individualized postprocessing with the SPM program 16. They reported concordance between VEEG findings and MRI signal abnormalities of up to 15.8%. In addition, signal changes outside the region of the electroclinical origin of seizures were observed in up to 42% of patients.

First, the analysis of these quantitative data obtained from MR sequences in our study was largely based on a comparison with the presumed EZ as determined by surface VEEG findings. This technique is associated with some limitations, but it provided the most appropriate localized data for these patients available at the time of the study. In addition to the difficulty of determining regionalization and lateralization in a significant number of patients, there is still the problem of the possibility of misleading localization of the EZ 17. This means that, in some patients, the signal abnormalities identified in postprocessing that are outside the regions presumably considered the EZ may reflect the actual epileptogenic focus.

It is also important to point out that the network involved in the seizure can be more widespread, probably explaining the widespread abnormalities observed in quantitative measurements 18,19. Despite the well-described relationship between FCD (the main cause of seizures in this group of patients) and the alterations found in the quantitative measures in epileptogenic regions, we cannot automatically relate abnormalities identified in postprocessing to the presence of an EZ.

It is worth noting that it is unclear whether a correlation between abnormalities evidenced by quantitative postprocessing methods, the electroclinical origin of seizures, and the resected region in patients who undergo epilepsy surgery without any visible epileptogenic structural alterations on conventional MRI has any influence on postoperative outcomes 7. However, there is ample literature on the significant benefits of the identification of potentially epileptogenic structural lesions on conventional MRI in the preoperative evaluation of candidates for surgery 6. Thus, we consider it very relevant that, even in a hospital recognized as a referral center for epilepsy surgery, we were able to reduce the number of patients classified as NLE when we used the location of abnormalities identified on postprocessing to guide visual inspection of conventional MRI. It is still important to emphasize that the patients analyzed in this study are part of a very challenging group, in which there are difficulties in the definition of the EZ even after multimodal (electrophysiological, functional and imaging) evaluation.

Indeed, the detection of potentially epileptogenic structural lesions improved in 5 out of 46 patients (10.9%). Two of these had been classified as NSLF by VEEG and could now be further investigated for EZ localization with a more precise, targeted approach. In one patient, quantitative findings were discordant with the purported electroclinical origin of the seizures. This disagreement did not cause postprocessing findings to be discarded, but it does suggest the need for greater caution in further investigations. In practice, this means that if these patients are to undergo surgery, they will benefit from the significant positive impact of having a lesional MRI 6.

Moreover, by adding the patients in the retrospective analysis, we included a total of eight patients (17.4%) who presented with some potentially epileptogenic abnormalities and were initially not characterized after visual evaluation guided by postprocessing and/or VEEG. In five of these patients, the 3D-FLAIR sequence provided significant benefits to the analysis, and in three patients, this sequence was the only one that allowed us to suspect FCD. These results are in line with those of Saini et al. 20, who concluded that this sequence should be included, especially when the MRI is considered negative and the patient is a candidate for surgery. This improvement in the diagnosis rate can be explained by the higher spatial resolution and the possibility of multiplanar reconstructions.

Finally, FLAIR-VBM has been recently used for the detection of cortical lesions in a group of patients with epilepsy and has presented superior results than T1-VBM 7,15. The main advantage of FLAIR-VBM over T1-VBM consists of the use of the T2 contrast typically found in FCD. Our study did not assess 3D-FLAIR because this technique was incorporated into the routine MRI protocol for epilepsy.

Other forms of evaluation of FLAIR and T1-VBM junction maps are also worth mentioning; for example, Wang et al. 21,22 used a morphometric analysis program (MAP) and found excellent correlations between FCD MAP+ regions. However, these studies were retrospective. Future prospective studies will be essential to test these and other quantitative techniques.

## CONCLUSION

Quantitative MRI evaluation of selected parameters through individualized postprocessing seems capable of identifying hidden structural alterations in patients with DRNFE. However, caution should be exercised when analyzing abnormalities identified through these techniques alone due to their apparently low specificity for EZ determination. These findings identified on postprocessing in this sample of patients generated several hypotheses; further research to test these hypotheses is warranted. In addition, as new techniques and sequences with higher spatial resolution become available, new studies should be carried out with the purpose of evaluating their utility and efficiency for the identification of epileptogenic structural lesions. Consequently, other methods, including invasive EEG recordings with stereo-EEG, may be performed to confirm that these findings indeed represent EZs.

Above all, our study showed the possibility of enhanced performance and detection of potentially epileptogenic lesions that had gone unnoticed on visual analysis of conventional MRI through targeted re-evaluation of such images guided by postprocessing. We also highlight the significant positive contribution of the 3D-FLAIR sequence in the detection of initially overlooked MRI lesions and reiterate that repeated visual analysis is an easy and readily available tool that should always be used while further research on these quantitative techniques is being conducted.

## AUTHOR CONTRIBUTIONS

Abud LG conceived and designed the study was responsible for the data collection, analysis and interpretation, statistical analysis, manuscript writing, manuscript critical revision and final approval. Velasco TR was responsible for data collection, analysis and interpretation, manuscript writing, critical revision and final approval. Salmon CEG was responsible for the analysis and interpretation, statistical analysis, manuscript writing, critical revision, and final approval. Sakamoto AC was responsible for the analysis and interpretation, and manuscript final approval. Abud TG was responsible for the manuscript critical revision and final approval. Pessini RA was responsible for the data collection and manuscript final approval. Abud DG and Leite JP were responsible for the manuscript critical revision and final approval. Santos AC conceived and designed the study and was responsible for the data collection, analysis and interpretation, manuscript writing, critical revision and final approval. All of the authors have read and approved the final version of the manuscript.

## Figures and Tables

**Table 1:** Characteristics of control groups and the parameters chosen after tests in each quantitative measure.

Quantitative measure	n	Median age (years)	Range (years)	SD (years)	Smoothing (mm)	*p*-value	Cluster of voxel
CThk	92 (27 men)	30.2	19-51	9.8	10	≤0.05 (FDR)	-
WGJS	92 (27 men)	30.2	19-51	9.8	8 FWHM	≤0.001 (uncorrected)	30
T2	53 (27 men)	30.9	18-51	9.1	6 FWHM	≤0.05 (FWE)	55
MTR	48 (30 men)	32.9	18-51	9.9	6 FWHM	≤0.05 (FWE)	30
MD	45 (28 men)	31.2	18-50	10.0	6 FWHM	≤0.05 (FWE)	40

CThk, cortical thickness; WGJS, white-gray matter junction signal; T2, T2 relaxometry maps; MTR, magnetization transfer rate; MD, mean diffusivity; FWHM, full width at half maximum; FDR, false discovery rate; FWE; familywise error.

**Table 2:** Quantitative MRI findings in 46 patients with drug-resistant focal epilepsy

Positive findings No. of patients/all patients with available data (%)	CThk	WGJS	MTR	T2	MD	Overall
In any lobe or region of the cerebral hemispheres	15/46 (32.6%)	17/46 (36.9%)	15/38 (39.5%)	20/41 (48.8%)	22/39 (56.4%)	39/46 (84.8%)
SLF corresponding to the presumed electroclinical source of the seizures	3/31 (9.7%)	3/31 (9.7%)	4/26 (15.4%)	7/28 (25.0%)	9/29 (31.0%)	16/31 (51.6%)
SLF not corresponding to the presumed electroclinical source of the seizures	8/31 (25.8%)	9/31 (29.0%)	9/26 (34.6%)	9/28 (32.1%)	15/29 (51.7%)	26/31 (83.9%)
Postprocessing failures	0	0	8/46 (17.4%)	5/46 (10.9%)	7/46 (15.2%)	16/46 (34.8%)

CThk, cortical thickness; WGJS, white-gray matter junction signal; T2, T2 relaxometry maps; MTR, magnetization transfer rate; MD, mean diffusivity; SLF, suspected location of epileptogenic focus.
